# BFVD—a large repository of predicted viral protein structures

**DOI:** 10.1093/nar/gkae1119

**Published:** 2024-11-22

**Authors:** Rachel Seongeun Kim, Eli Levy Karin, Milot Mirdita, Rayan Chikhi, Martin Steinegger

**Affiliations:** Interdisciplinary Program in Bioinformatics, Seoul National University, Seoul, Republic of Korea; School of Biological Sciences, Seoul National University, Seoul, Republic of Korea; ELKMO, Copenhagen, Denmark; School of Biological Sciences, Seoul National University, Seoul, Republic of Korea; Institut Pasteur, Université Paris Cité, G5 Sequence Bioinformatics, Paris, France; Interdisciplinary Program in Bioinformatics, Seoul National University, Seoul, Republic of Korea; School of Biological Sciences, Seoul National University, Seoul, Republic of Korea; Institute of Molecular Biology and Genetics, Seoul National University, Seoul, Republic of Korea; Artificial Intelligence Institute, Seoul National University, Seoul, Republic of Korea

## Abstract

The AlphaFold Protein Structure Database (AFDB) is the largest repository of accurately predicted structures with taxonomic labels. Despite providing predictions for over 214 million UniProt entries, the AFDB does not cover viral sequences, severely limiting their study. To address this, we created the Big Fantastic Virus Database (BFVD), a repository of 351 242 protein structures predicted by applying ColabFold to the viral sequence representatives of the UniRef30 clusters. By utilizing homology searches across two petabases of assembled sequencing data, we improved 36% of these structure predictions beyond ColabFold’s initial results. BFVD holds a unique repertoire of protein structures as over 62% of its entries show no or low structural similarity to existing repositories. We demonstrate how a substantial fraction of bacteriophage proteins, which remained unannotated based on their sequences, can be matched with similar structures from BFVD. In that, BFVD is on par with the AFDB, while holding nearly three orders of magnitude fewer structures. BFVD is an important virus-specific expansion to protein structure repositories, offering new opportunities to advance viral research. BFVD can be freely downloaded at bfvd.steineggerlab.workers.dev and queried using Foldseek and UniProt labels at bfvd.foldseek.com.

## Graphical Abstract

**Graphical Abstract:**
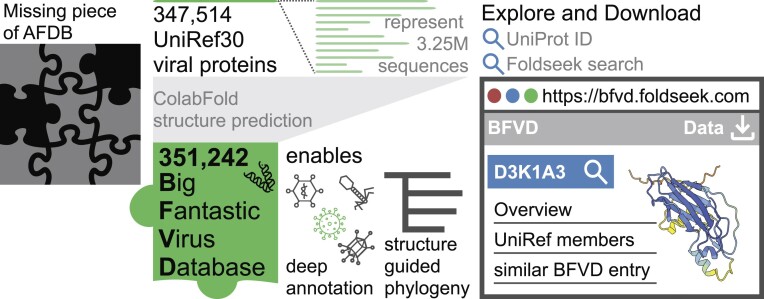

## Introduction

Viruses are infectious agents that invade host cells, exploiting their biological machinery for replication. They mutate rapidly, evading existing treatments and immunity, thus posing a persistent threat to public health (1). Their huge genetic diversity, often reflected in <30% amino acid sequence identity between newly discovered viruses and known ones, presents challenges for sequence-based annotation and classification (2,3). In contrast, due to their direct effect on function, protein structures tend to be more conserved, which can be used for studying viral mechanisms (4–6). Therefore, the availability of viral protein structures is critical for viral annotation through the detection of structural similarities.

Recent advancements in computational protein structure prediction (7–10) have made hundreds of millions of protein structures available through repositories like the AlphaFold Protein Structure Database (AFDB) (11,12) and the ESM Atlas (8). These repositories have been transformative for studying the function of many proteins and protein families as a whole (13,14). Using the AFDB has also contributed to the study of viruses, e.g. by improving the annotation of metagenomic bacteriophages (15) and by revealing viral proteins acquired from their metazoan host (16).

However, leveraging these vast resources for virus research remains limited as the AFDB excludes viral proteins and the ESM Atlas lacks taxonomic information, making it difficult to identify viral proteins. Consequently, studying viral structures still relies on in-house prediction of protein structures [e.g., (17,18)], which is a time- and resource-consuming task. Recently, notable efforts have been made to minimize the gap in available viral structures, including the release of HerpesFolds (19), which offers high-quality structure predictions for all nine human herpesviruses. In the same vein, Nomburg *et al.* (20) predicted 67 715 protein structures from 4463 species of eukaryotic viruses, creating a resource—hereafter referred to as ‘Nomburg24’—which has been integrated into the viral repository ViralZone (21). Despite making milestone contributions to the study of viruses, HerpesFolds and Nomburg24 are limited to eukaryotic viruses and do not cover other viral clades.

Here, we focused on the viral fraction of UniProt (22) by examining its 30% sequence-identity clusters from UniRef30 (23,24). We then predicted the protein structures of 351 242 cluster representatives of viral origin, whose clusters jointly consisted of over three million protein sequences. We improved the quality of 36% of these predictions by mining petabases of assembled sequence reads in Logan (25), resulting in over 99 million similar sequences, added to the input for structure prediction. This effort resulted in BFVD, the largest repository of predicted viral structures to date. We show that BFVD contains highly diverse structures of various viral kingdoms, covering more viral variance than existing resources. We then demonstrate using BFVD for discovering similar structures to bacteriophage proteins, which could not be annotated based on their sequences, highlighting its utility tailored to viral research.

## Materials and methods

### Preparing UniRef sequences for BFVD

The clustering at 30% pairwise sequence identity of UniProt (22) sequences release 2023_02 were extracted from the UniRef30 ColabFold database (10,23,24) at https://colabfold.mmseqs.com. This dataset has 36 293 491 clusters, of which 347 514 have a viral sequence representative, as evident by their assigned NCBI taxonomic identifier (taxid), which is a descendant of taxid 10239 (‘Viruses’). These clusters jointly contained 3 248 875 protein sequences and their 347 514 representatives were collected for the construction of BFVD. To limit the computational demand of the structure prediction step, we ensured that no sequence exceeded 1500 residues in length. To that end, the 3002 sequences longer than this threshold were split into consecutive, non-overlapping fragments, as follows. Let *L* be the length of such a sequence, then *F* = ⌈*L*/1500⌉ was the number of fragments it was divided to, and in each fragment there were *R* = ⌊*L*/*F*⌋ residues (except for the last, which may have been longer due to including the remainder). This splitting resulted in 6732 sequence fragments, two of which contained only ’X’ in their amino-acid sequence and were excluded, leaving 6730. BFVD was thus constructed from a total of 351 242 viral sequences.

### Taxonomic composition of BFVD

**Figure 1.**
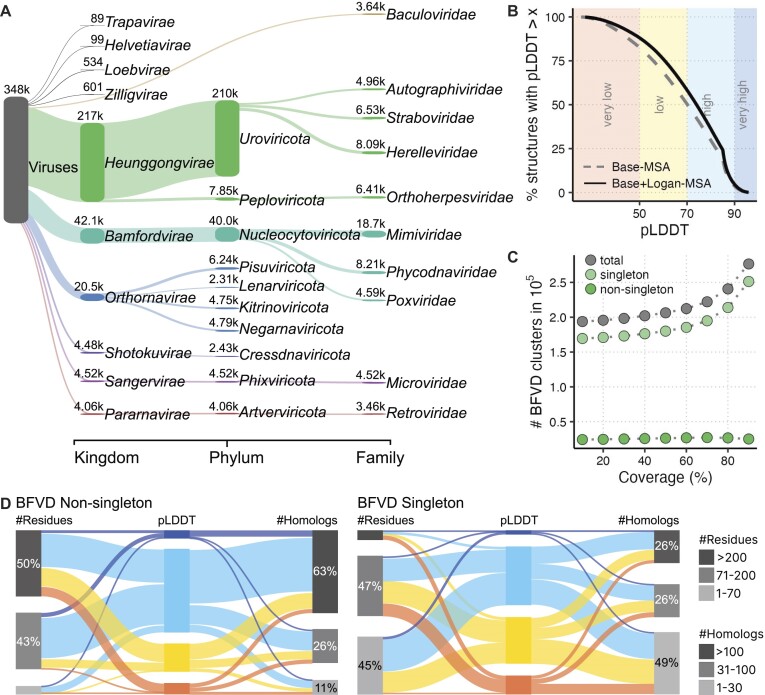
BFVD composition and cluster analysis. **(A)** BFVD taxonomic composition. Shown at each rank are the 10 most abundant taxa. **(B)** Cumulative distributions of pLDDT scores among BFVD’s predicted structures before Logan’s additional homologs (dashed) and after (full line). Over a half are highly confident. **(C)** Structural redundancy reduction using Foldseek *cluster*. The number of structural clusters, especially singletons, increases with the value of the coverage parameter, though moderately until 70%. **(D)** An alluvial plot of BFVD structures clustered at 70% coverage. pLDDT intervals indicated in color as in (B). Non-singleton proteins (left panel) are longer (left column) and have more homologs in their MSAs (right column) than singleton proteins (right panel).

The taxid for each BFVD sequence was retrieved from UniProt and its full lineage—from NCBI (26). The Sankey plot based on this information (Figure 1A) was generated with Pavian (27). At each taxonomic rank, only the ten most abundant taxa were included in the plot.

### Structure prediction

Each of the 351 242 viral UniRef30 clusters is associated with two summary sequences: a representative and a consensus. The former is a biological sequence, belonging to some virus, and the latter is the computational summary of the UniRef30 cluster. For the construction of BFVD we used both as follows. Each consensus was used to query for homologs using *colabfold_search* utilizing MMseqs2 (version ede0be1) (28) against the ColabFold (10) reference databases ‘uniref30_2022’ and ‘colabfold_envdb_202108’ and for computing a multiple sequence alignment (MSA), denoted here as the ‘base-MSA’. Next, the structure of each representative sequence was predicted based on its corresponding base-MSA using ColabFold v.1.5.2 (10) and the AlphaFold2 model with default parameters, except for ‘- -num-models’ and ‘- -stop-at-score’, which were set to 3 and 85, respectively. For each representative, the best-ranking structural model according to the pLDDT score was kept. Predicting these structures took approximately one GPU-year of compute time spread across several weeks on 4 to 14 NVIDIA RTX A5000 GPUs.

### Search for homologs in Logan

The 175 788 viral UniRef30 consensus sequences which had fewer than 30 homologs in their base-MSA (see section ‘Structure prediction’) were used to construct an amino-acid reference database using DIAMOND (29) v2.1.9 *makedb* command. Next, each of Logan’s (25) contigs V1 (https://github.com/IndexThePlanet/Logan/blob/main/Stats-v1.md) was searched against this reference database using DIAMOND v2.1.9 *blastx* command (parameters: -c 1 - -masking 0 - -sensitive -s 1 - -evalue 1e-8 -k 1). The script to distribute the search was obtained from https://gitlab.pasteur.fr/rchikhi_pasteur/logan-analysis. This search detected regions within 989 683 364 Logan amino-acid sequences, matching the BFVD. The redundancy of these regions was reduced by clustering them at 90% sequence-identity using the *easy-linclust* module of MMseqs2-Linclust (30) (version: 15.6f452; parameters: - -min-seq-id 0.9 -c 0.9 - -cov-mode 1 - -kmer-per-seq 80), resulting in a set of 99 115 059 Logan representative sequences. Next, the 175 788 BFVD sequences were queried against the Logan redundancy-reduced set using MMseqs2 (28) *search* (version: 15.6f452; parameters: - -max-seqs 10000 -s 7 -e 0.1) followed by *result2msa* (parameters: - -msa-format-mode 6). This resulted in Logan-MSAs, where the Logan homologs were aligned to the BFVD consensus sequence. Each Logan-MSA was then appended to its corresponding base-MSA, creating joint MSAs, which were provided to ColabFold through the ‘custom MSA’ option (see Box 3, (31)). Using these, ColabFold repredicted the structures of the 175 788 UniRef30 representatives, as described in the section ‘Structure prediction’. These predictions replaced the ones based only on base-MSAs in the final set of BFVD structures.

### Structural clustering of BFVD

The redundancy reduction of BFVD was performed as described in ‘Results’ using the Foldseek v.9.427df8a *easy-cluster* module with coverage threshold of 70%. The same Foldseek version and the module *search* were used for querying BFVD against itself for the web server using default parameters and an E-value threshold of 0.01.

### Comparing BFVD to AFDB50 and PDB100

Foldseek (32) v.9.427df8a *easy-search* module was used to query the structures of BFVD against those of AFDB50 (12,14) and of PDB100 (32,33). The option ‘- -greedy-best-hits’ was enabled to cover each query with the best match(es) to AFDB50 or PDB100. In case several matches were found, the one with the highest query-normalized TM-score (34) was selected. For residue-wise assessment, the LDDT values computed by Foldseek for each alignment were extracted.

### Structural clustering of BFVD and Nomburg24

The joint clustering of 351 242 BFVD structures and 67 715 Nomburg24 structures was performed as described in ‘Results’ following the steps of the Foldseek v.9.427df8a *easy-cluster* module with a coverage threshold of 70%. This resulted in 32 755 non-singleton clusters, of which 22 700 (consisting of 97 365 structures) were unique to BFVD and 875 (2816 structures)—unique to Nomburg24 and the rest had a structure from both databases.

### Bacteriophage annotation

We followed the bacteriophage annotation pipeline by Say *et al.* (15), with few modifications, as described in the following.

*Obtaining and assembling the GAC6 sample*. The dataset for GAC6 was obtained from the European Nucleotide Archive accession PRJEB49151 (https://www.ebi.ac.uk/ena/browser/view/PRJEB49151) by selecting ‘t3_may7-2020’ in the ‘sample title’ field. The following steps were performed as described by Say *et al.*, using the same parameters: base calling using Guppy v.6.3.8, filtering using NanoFilt (35) v.2.8.0 and assembly using Flye (36) v.2.9.3-b1797. Unlike Say *et al.*, we omitted the secondary assembly step and directly extracted circularized assemblies with a minimum coverage of ten.

*Read mapping and polishing*. Reads were mapped to each assembly using Minimap2 (37) v2.24, filtered by Gerenuq (https://doi.org/10.5281/zenodo.5119771) v.0.2.3 and polished by Minipolish (38) v.0.1.3, using the same parameters as Say *et al.*

*Bacteriophage detection*. Following Say *et al.*, the polished assemblies were annotated with Bakta (39) v.1.5.1 and then with INHERIT (40), retaining only assemblies annotated as bacteriophages.

*Structure prediction*. In all, 1329 proteins on 17 contigs were annotated as ‘bacteriophage’ at the end of the last step. Like Say et al., we predicted the structures of these sequences using ColabFold v.1.5.5 with the same arguments. We retained for each sequence the best-ranking structural model according to the pLDDT score.

*Bakta-hypothetical and Foldseek search*. Like Say et al., we counted proteins which Bakta did not annotate and labeled as ‘hypothetical’ as Bakta-hypothetical. The predicted structures of all Bakta-hypothetical proteins (1221) were queried using Foldseek v.9.427df8a *easy-search* module against the AFDB, with an E-value cutoff of 0.001, as in Say *et al.* In addition, they were queried against BFVD and Nomburg24. The search results against the BFVD and the AFDB were also merged and examined together.

## Results

### Construction of the Big Fantastic Virus Database (BFVD)

We first collected the representative protein sequences from UniRef30’s viral clusters, covering major viral clades (Figure 1A). To limit the computational demand of structure prediction, we split 3002 sequences longer than 1500 residues (<1% of all) into 6730 sequence fragments. These fragments and the other sequences were provided as queries to ColabFold. After collecting homologs for each query, ColabFold computed its associated base multiple sequence alignment (base-MSA, see Methods) and predicted its structure. This resulted in 351 242 viral protein structures with a median predicted Local Distance Difference Test (pLDDT) of 70.18 and an interquartile range (IQR) of 55.8–82.2, indicating medium confidence (Figure 1B, dashed). As previously reported, prediction accuracy is negatively affected by an insufficient number of homologs in the MSA used for structure prediction, especially when there are fewer than 30 sequences (7,41). Indeed, among the low-confidence structure predictions (pLDDT < 50), the majority (72%) had fewer than 30 homologs in their base-MSA.

### Logan homologs improve BFVD’s structures

Therefore, we focused on 175 788 BFVD structures, which had shallow base-MSAs (<30 homologs) and used Logan (25), a recently-released assemblage of the Sequence Read Archive (42), to seek additional homologs for them, in two petabases of assembled contigs. Using DIAMOND, we detected over 989 million sequences in Logan similar to the shallow BFVD set. We reduced their redundancy by clustering them at 90% sequence-identity with MMseqs2-Linclust, keeping ca. 99 million Logan representatives. We then used MMseqs2 to search the Logan representatives and compute Logan-MSAs for the 175 788 BFVD sequences. Finally, we appended the Logan-MSAs to their corresponding ColabFold base-MSAs, resulting in substantially deeper joint MSAs (92.4 homologs on average (median: 18), compared to 7.4 (median: 4) without the Logan addition). Repredicting the structures using the joint MSAs improved in turn the quality of predictions for 35.6% of the BFVD structures, increasing the overall median of 70.18 pLDDT (IQR: 55.8–82.2) to 74 (IQR: 60.7–84.7) (Figure 1B).

### Viral coverage of BFVD

To check BFVD’s span, we predicted the structures of entire proteomes of seven highly varied viruses (differ by DNA/RNA, single/double stranded, genome size and host) using ColabFold (Supplementary Figure 1). We then used Foldseek to query these proteomes against BFVD and found that six out of seven viruses had a match in BFVD for most (92–100%) of their predicted proteins (Supplementary Figure 1). One proteome, that of SARS-CoV-2, had BFVD matches for only 65% of its 23 predicted structures. However, examining the unmatched proteins revealed they were generally short and unstructured.

### BFVD structural clustering analysis

Next, we reduced structural redundancy in BFVD by using Foldseek *cluster* to group together similar structures. We first studied the number of clusters obtained under different values of the Foldseek *cluster* coverage parameter (Figure 1C). This parameter determines the minimal bidirectional coverage between a cluster representative and each cluster member, with lower values being more permissive. The total number of clusters increased from 193 787 to 276 477 following an increase in the coverage parameter. Furthermore, over 48% of the BFVD structures did not cluster and remained as singletons even at the lowest coverage threshold.

To investigate possible reasons why so many BFVD protein structures failed to cluster, we focused on the clustering with 70% coverage cutoff, below which the number of singletons plateaued (Figure 1C). We compared BFVD structures clustered as non-singletons and as singletons (Figure 1D) by three attributes: their lengths, the number of homologs in their structure-prediction MSAs, and their pLDDT scores. We found that singleton structures were shorter than non-singleton ones (median and average number of residues: 75 and 99, compared to 202 and 307.9). Focusing on the shortest structures (≤70 residues), we found that 89.3% of them were singletons. Unlike longer structures, only 2.2% of the shortest structures exhibited low confidence scores (pLDDT < 50). This is consistent with a previous report of high pLDDTs in sequences shorter than 100 residues (43). Singleton protein structures also tended to have shallower MSAs, with an average of 305 homologs (median: 32), while non-singleton protein structures had an average of 1255 (median: 198). Put together, the high abundance of structural singletons in BFVD is likely driven by the short length and the limited number of homologs of singleton proteins.

### A web server to explore BFVD

**Figure 2.**
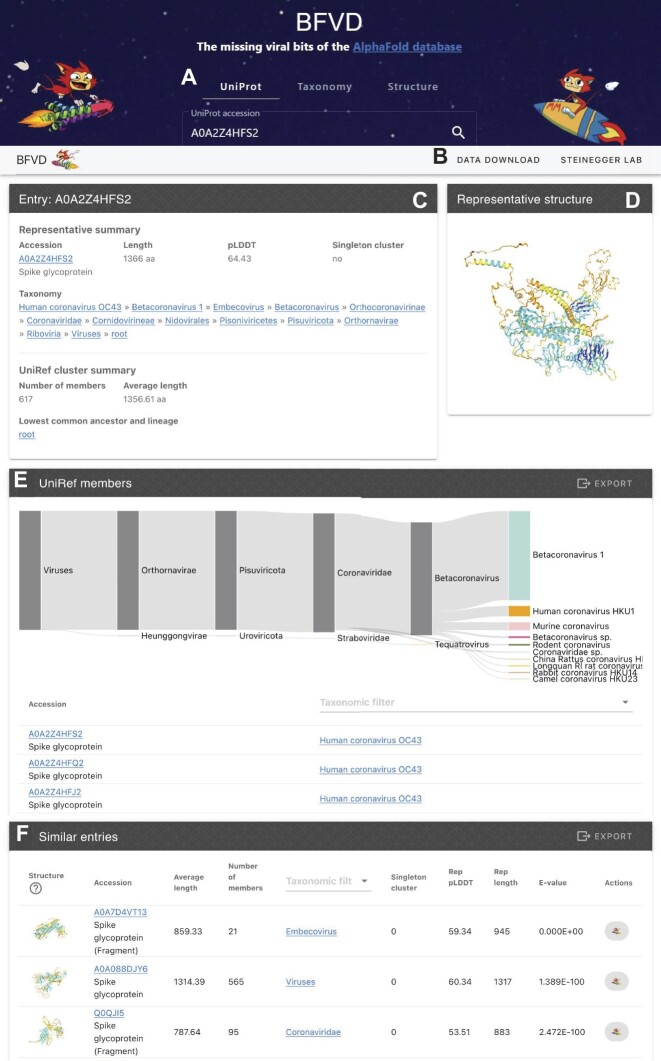
The BFVD web server. **(A)** Users can query BFVD by UniProt identifiers and taxonomic labels as well as Foldseek structural search. **(B)** Link to data download. **(C)** Overview of a BFVD entry and its UniRef members. **(D)** Interactive structure visualization. **(E)** Taxonomic distribution of UniRef cluster members. **(F)** BFVD entries similar to the current entry, as determined by a Foldseek all-vs.-all search. Structures can be superposed to the current entry using TM-align (34) by clicking on the structure visualization (left) or sent to the Foldseek webserver for search against various structure databases.

Building on top of our previously released web server for the AFDB clusters (14), we set up a web server at bfvd.foldseek.com to allow exploring the BFVD structures in the context of the over three million UniRef sequences they represent (Figure 2). For each BFVD structure, the web server can display its 3D model, inform about its prediction quality, list the entries in its UniRef30 cluster, present its associated taxonomic labels (and host, where available), and indicate whether it is a BFVD structural singleton or not. In addition, we queried the BFVD structures against themselves using Foldseek *search*, allowing the web server to link from each BFVD structure to all structurally similar BFVD entries. Owing to the integration of these various annotations, the web server enables users to query BFVD by providing either a UniProt accession, or a taxonomic label, or a protein structure. If a structure is provided as query, the web server will search the BFVD structures using Foldseek. As part of this study, BFVD was also added as a reference database to the Foldseek webserver at search.foldseek.com.

### BFVD’s structural novelty compared to existing resources

**Figure 3.**
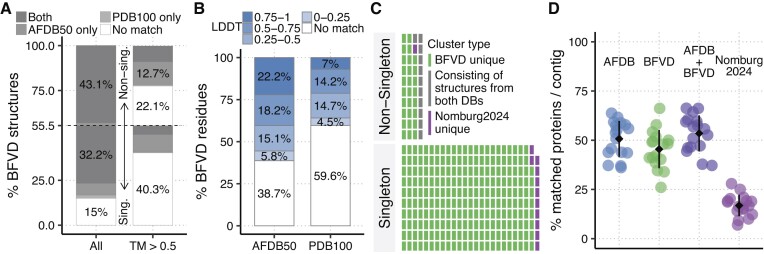
BFVD compared to other protein structure repositories. (A, B) Foldseek comparison of BFVD to AFDB50 and PDB100 reveals its uniqueness. **(A)** Match fractions are presented separately by a dashed line for BFVD singletons (ca. 55% of its structures) and non-singletons (45%). Ca. 15% of BFVD’s structures, all singletons, do not match AFDB50/PDB100 (left). Excluding low-similarity matches, ca. 62% of all BFVD’s structures cannot be matched (right). **(B)** Also on the residue-level, low similarity is observed against AFDB50 (left) and PDB100 (right). **(C)** Joint clustering of BFVD and Nomburg24. Each cell represents 800 clusters. BFVD’s structures are found in most clusters (96%), indicating its broad structural repertoire compared to Nomburg24. **(D)** Analysis of GAC6 bacteriophage contigs as in Say *et al.* Most proteins on each contig (79.4–98.6%) remained unannotated using the sequence-based method Bakta. Foldseek matches large fractions of Bakta-hypothetical proteins with a structural hit using various reference databases (colors). BFVD matches comparable fractions to the AFDB, while being much smaller. Depicted are the matched fractions on the 17 contigs (points), standard deviations (bars) and averages (diamonds).

To assess the novelty of BFVD, we used Foldseek to compare its structures to those of two major resources: AFDB50, a clustered version of the AFDB, consisting of 52 million cluster representatives, and the 100% sequence identity clustered Protein Data Bank (PDB100) with 279 193 entries (Figure 3A). We found that ca. 15% of the structures, all singletons, were unique to BFVD, matching neither AFDB50, nor PDB100. An additional 10%, mostly singletons, matched only one of these databases. Furthermore, applying a cutoff on the quality of the match (TM score ≥ 0.5) revealed that only 38% of the BFVD structures matched any of the two databases. We then evaluated BFVD’s residue-level similarity to these databases by retrieving the alignment LDDT values computed by Foldseek for each match (Figure 3B). We found that ca. 39% and 60% of the BFVD residues could not be aligned to AFDB50 and to PDB100, respectively. Additional fractions of ca. 6% and 5% could be matched to these databases only with a poor score (LDDT < 0.25). These results indicate that BFVD offers a unique opportunity to explore viral diversity that existing databases do not capture.

Recently, 67 715 protein structure predictions from eukaryotic viruses were made available in Nomburg24. To delineate the structural variation of BFVD and Nomburg24, we applied Foldseek *cluster* to the joint set of their structures. This resulted in 32 755 non-singleton clusters, consisting of 218 914 structures, and in 200 043 singleton clusters (8913 from Nomburg24 and 191 130 from BFVD) (Figure 3C). Considering each of these 232 798 clusters as a putative structural class, we found that BFVD covered about 96% of all classes by having a structure in nearly all (97%) non-singleton clusters and producing the most singletons. In contrast, Nomburg24 covered only 8% of all classes by having a structure in 31% of all non-singleton clusters and producing substantially fewer singletons.

### Case study: BFVD for studying bacteriophage proteins

To demonstrate BFVD’s utility, we repeated and extended a part of a recent study by Say *et al.* (15) that annotated putative bacteriophages within metagenomically assembled contigs from wastewater. Say *et al.* developed a pipeline for enhanced annotations by integrating structural information from the AFDB with sequence data. Here, we applied the steps of their pipeline to one of the samples from their study: the Granulated Activated Carbon sample 6 (GAC6). In addition to using the AFDB, we included BFVD and Nomburg24 as reference databases for structural similarity search (Figure 3D). Like Say *et al.*, we found that the sequence-similarity based tool Bakta (39) matched on average 8% of the putative bacteriophage proteins on each contig with non-hypothetical labels, considering the rest as Bakta-hypothetical. As in their study, Foldseek with the AFDB as reference found on average a structural match for 51% of the proteins in the Bakta-hypothetical fraction. By using BFVD, we could find structural matches for a comparable average of 45%, despite the tremendous size difference between the AFDB and BFVD. However, combining the search results against the AFDB and BFVD only marginally increased the fractions of matched structures. This suggests that the AFDB likely includes some BFVD bacteriophage structures indirectly, through prophages embedded in bacterial genomes covered by the AFDB. Compared to the AFDB and BFVD, Nomburg24 matched lower fractions of the Bakta-hypothetical proteins, likely due to its focus on eukaryotic viruses.

## Discussion

We presented BFVD, a database of 351k predicted protein structures from the viral fraction of UniRef30 and we improved over a third of its predictions by integrating 99 million homologs identified through a petabase-scale sequence search. When clustering its structures, we found that BFVD had a high prevalence of singletons (55%), compared to AFDB50 (25% of all structures) (14). Investigating possible reasons, we found that singletons tend to be structures predicted from shorter proteins with fewer homologs, compared to non-singletons. Singletons should thus be treated with caution as they may not represent valid structural classes, but rather the result of poor structure prediction due to shallow MSAs or the presence of disordered regions. We then showed that BFVD is unique and substantially different from the AFDB and the PDB as well as Nomburg24. BFVD is more comprehensive than Nomburg24, as revealed by the analysis of their joint clustering and by their utility for matching bacteriophage proteins. In this bacteriophage case study, BFVD achieved comparable performance to the AFDB, effectively replacing the need for its 214 million entries with only 351k structures. This highlights the value of BFVD for virus-specific studies, offering a compact but comprehensive resource, tailored to their needs. Moreover, since the entries in BFVD originate from UniProt, users can easily augment them with UniProt’s taxonomic and functional annotations to enhance the study of viral biology. BFVD’s structures can be used with current tools like Foldseek and its webserver, in BFVD’s designated web server, as well as newly developed ones, like the multiple structure aligner FoldMason (44) to shed new light on viral function and evolution. Looking ahead, we aim to expand BFVD by predicting viral multimer structures, taking advantage of their compact genome size, and making them searchable using Foldseek-Multimer (45).

## Supplementary Material

gkae1119_Supplemental_File

## Data Availability

All metadata, predicted structures (available as a tar file of PDBs), as well as the Foldcomp (46) and Foldseek databases, can be freely downloaded from bfvd.steineggerlab.workers.dev. Analysis scripts are available at github.com/steineggerlab/bfvd-analysis. The webserver code is available at github.com/steineggerlab/afdb-clusters-web/tree/bfvd. Source Code and Data have been archived in Zenodo at https://doi.org/10.5281/zenodo.13992244 and https://doi.org/10.5281/zenodo.13993144, respectively.
