# Supplementary material for: BFVD—a large repository of predicted viral protein structures
Source: Nucleic Acids Res. 2024 Nov 22;53(D1):D340–7. doi: 10.1093/nar/gkae1119 (PMC11701548; doi:10.1093/nar/gkae1119)
Supplement: gkae1119_Supplemental_File [file gkae1119_supplemental_file.pdf]

Published online DD MM YYYY *Nucleic Acids Research, YYYY, Vol. xx, No. xx* **1–1**
doi:10.1093/nar/gkn000

Supplementary: BFVD - a large repository of predicted viral protein structures

Rachel Seongeun Kim [1,2], Eli Levy Karin [3], Milot Mirdita [2], Rayan Chikhi [4] and Martin Steinegger [1,2,5,6*]

[1]Interdisciplinary Program in Bioinformatics, Seoul National University, Seoul, Republic of Korea
[2]School of Biological Sciences, Seoul National University, Seoul, Republic of Korea
[3]ELKMO, Copenhagen, Denmark
[4]Institut Pasteur, Université Paris Cité, G5 Sequence Bioinformatics, Paris, France
[5]Institute of Molecular Biology and Genetics, Seoul National University, Seoul, Republic of Korea
[6]Artificial Intelligence Institute, Seoul National University, Seoul, Republic of Korea

Received YYYY-MM-DD; Revised YYYY-MM-DD; Accepted YYYY-MM-DD

*To whom correspondence should be addressed. Email: martin.steinegger@snu.ac.kr

© YYYY The Author(s)
This is an Open Access article distributed under the terms of the Creative Commons Attribution Non-Commercial License (http://creativecommons.org/licenses/by-nc/2.0/uk/) which permits unrestricted non-commercial use, distribution, and reproduction in any medium, provided the original work is properly cited.

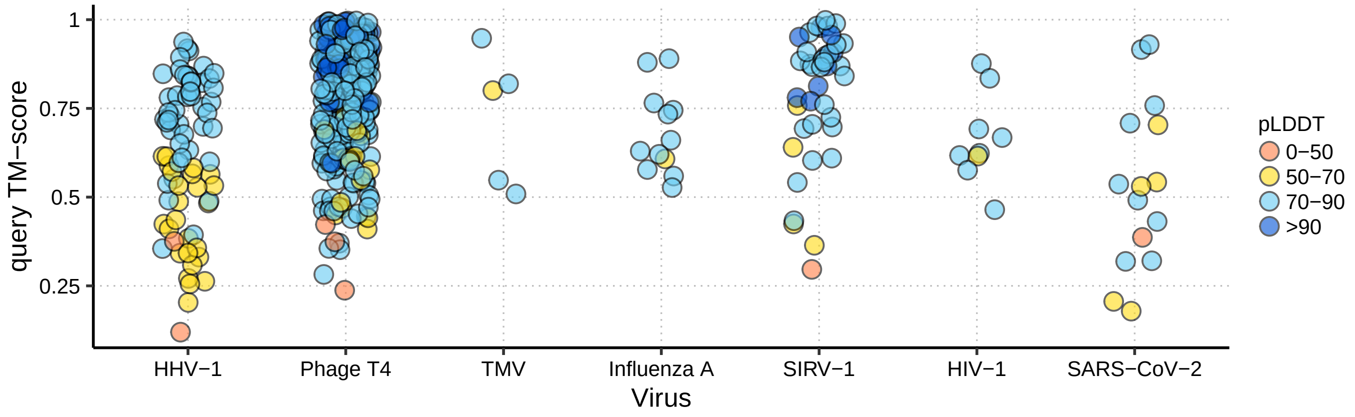

Virus	# split proteins	# matched (E < 0.01)	# not matched	% matched
Human herpesvirus 1 (strain 17)	75	74	1	98.67
Enterobacteria phage T4	273	251	22	91.94
Tobacco mosaic virus (strain vulgare)	4	4	0	100
Influenza A virus (strain A/Goose/Guangdong/1/1996 H5N1 Gs/Gd)	13	12	1	92.31
Sulfolobus islandicus rod-shaped virus 1	43	40	3	93.02
Human immunodeficiency virus type 1 group N (isolate YBF30)	9	9	0	100
Severe acute respiratory syndrome coronavirus 2	23	15	8	65.22

Supplemental Figure 1. Analysis of BFVD span by varied full proteomes. Seven full proteomes (UniProt accessions: UP000009294 for HHV-1, UP000009087 for Phage T4, UP000000522 for TMV, UP000131152 for Influenza A, UP000002270 for SIRV-1, UP000007420 for HIV-1 and UP000464024 for SARS-CoV-2) of highly variable (DNA vs. RNA, single/double strand, genome size, host) viruses were downloaded from the UniProt website on October 2024 using ‘wget’ commands. Four of the proteins were longer than 1,500 amino-acids and were therefore split into 13 fragments, as described in “Materials and Methods” for BFVD sequences. Next, 440 structures were predicted from these proteomes using ColabFold (version: v.1.5.2; parameters: the AlphaFold2 model with default parameters, except for ‘--num-models’ and ‘--stop-at-score’, which were set to 3 and 85). We then used Foldseek (version: v.9.427df8a; parameters: default, except ‘--format-output’ which was set to include query TM-scores: ‘qtmscore’) to query the structures of the proteomes against BFVD and found that six out of seven viruses had a match in BFVD for most of their predicted proteins (see **%matched** in table). Furthermore, well predicted proteins (high pLDDT; shades of blue in plot) were more likely to have higher query TM-scores. We then inspected the eight protein structures of SARS-Covid-2, which could not be matched by the BFVD. We found that they were either very short (P0DTD8: 43 amino-acids, P0DTG0: 57), unstructured (P0DTC6, P0DTF1: lacks a stable, globular structure), only contain one very simple domain (A0A663DJA2) or haven’t been experimentally-solved, suggesting they may not have a stable structure (P0DTG0, P0DTG1).
